# Association of *FTO* and *ADRB2* gene variation with energy restriction induced adaptations in resting energy expenditure and physical activity^☆^^☆☆^^☆☆☆^

**DOI:** 10.1016/j.gene.2019.100019

**Published:** 2019-05-18

**Authors:** Stefan G.J.A. Camps, Sanne P.M. Verhoef, Freek G. Bouwman, Edwin C.M. Mariman, Klaas R. Westerterp

**Affiliations:** a Maastricht University, Department of Human Biology, Nutrition and Toxicology Research Institute Maastricht (NUTRIM), 6200, MD, Maastricht, the Netherlands; b Clinical Nutrition Research Centre (CNRC), Singapore Institute for Clinical Sciences (SICS), Agency for Science, Technology and Research (A*STAR) and National University Health System (NUHS), Centre for Translational Medicine, 14 Medical Drive #07-02, MD6 Building, Yong Loo Lin School of Medicine, Singapore 117599, Singapore

**Keywords:** *ADRB2*, β2-adrenergic receptor, BMI, body mass index, FM, fat mass, *FTO*, fat mass and obesity associated, FFM, fat-free mass, GLM, general linear modelling, *MC4R*, melanocortin 4 receptor, *PPARD*, peroxisome proliferator-activated receptorδ, *PPARGC1A*, peroxisome proliferator-activated receptorγ coactivator-1α, REE, resting energy expenditure, REEm, resting energy expenditure, measured, REEp, resting energy expenditure, predicted, SNPs, single nucleotide polymorphisms, VLED, very low energy diet, Genetic predisposition, Metabolic adaptation, Adaptive thermogenesis, Weight loss, Energy balance

## Abstract

**Background:**

Energy restriction induces adaptations in resting energy expenditure (REE) and physical activity; inter-individual variability could be ascribed to genetic predisposition.

The aim was to examine if changes in REE and physical activity as a result of weight loss were affected by candidate single nucleotide polymorphisms (SNPs).

**Methods:**

148 subjects (39 men, 109 women), mean ± SD age: 41 ± 9 year; body mass index (BMI): 31.9 ± 3.0 kg/m^2^, followed a very low energy diet for 8 weeks. SNPs were selected from six candidate genes: *ADRB2*, *FTO*, *MC4R*, *PPARG2*, *PPARD* and *PPARGC1A*. REE (ventilated hood) and physical activity (tri-axial accelerometer) were assessed before and after the diet. General linear modelling included gender, age and additional relevant covariates for all parameters.

**Results:**

The heterozygotic genotype of *FTO* was associated with a higher amount of physical activity (1.71 Mcounts/d; CI 1.62–1.81) compared to the homozygotic major genotype (1.50 Mcounts/d; CI 1.40–1.59) (P < 0.001) while the homozygotic risk allele genotype was not different (1.56 Mcounts/d; CI 1.39–1.74) at baseline; moreover, a similar pattern was observed after energy restriction. Carrying the homozygotic minor genotype of *ADRB2* was associated with a larger decrease in REE (P < 0.05) and greater adaptive thermogenesis (P < 0.05) after weight loss.

**Conclusion:**

Carrying the minor *ADRB2* allele homozygous was associated with a larger diet induced metabolic adaptation in energy expenditure and suggest a central role for reduced lipid mobilization. Carrying the risk allele of *FTO* homozygous was not associated with lower physical activity at baseline or after weight loss. Heterozygous carriers of one *FTO* risk allele showed greater physical activity before and after weight loss which might protect them in part from the higher obesity risk associated with *FTO*.

## Highlights

• Variants of the *ADRB2* gene were associated with metabolic adaptation and suggest a central role for lipid mobilization.
• Carrying two *FTO* risk alleles was not associated with lower physical activity at baseline or after weight loss.
• Carrying one *FTO* risk allele was linked to more physical activity which may moderate the *FTO* related obesity risk.

## 1 Introduction

The increasing prevalence of obesity and its comorbidities is one of the major health problems in our modern world (Catenacci et al., 2009). Weight loss strategies may target both sides of the energy balance to induce a caloric deficit, mathematically a simple concept, though the success of long-term weight loss maintenance is low (Kraschnewski et al., 2010; Wing and Phelan, 2005). Part of the low success rate can be explained by the human body's adaptive biological response to weight loss as reviewed by MacLean et al. (Maclean et al., 2011) and further summarized by Mariman as a network of adaptations and physiological changes resulting in resistance for further weight loss and an energy gap promoting weight regain (Mariman, 2012). As part of a plethora of adaptations during energy restriction, resting energy expenditure (REE) and physical activity are also affected. Studies performed in lean and obese subjects have shown significant reductions in REE during and shortly after weight loss to values below predictions based on weight loss and body composition changes (Leibel et al., 1995a; Doucet et al., 2001; Dulloo and Jacquet, 1998; Schwartz and Doucet, 2010; Tremblay and Chaput, 2009; Weyer et al., 2000; Camps et al., 2013; Martin et al., 2007). The decrease in REE beyond what can be predicted by the loss of fat-free mass (FFM) and fat mass (FM), is defined as adaptive thermogenesis. In addition, several studies have demonstrated a decrease in physical activity and activity induced energy expenditure (AEE) as a result from weight loss (Martin et al., 2007; Camps et al., 2013; de Groot et al., 1989; Velthuis-te Wierik et al., 1995; Redman et al., 2009; Bonomi et al., 2013). These two metabolic adaptations can limit weight loss and can be important factors that compromise the maintenance of a reduced body weight.

Individuals respond differently to environmental weight gain risk factors and this inter-individual variation can partly be explained by genetics. Family and twin studies have shown a 40–70% genetic contribution in the susceptibility to develop obesity (Loos and Bouchard, 2003). The number of genetic loci that have been associated with obesity related traits is still increasing (Li et al., 2010) and the small fraction of the total variation that is explained by the individual loci and inconsistent findings, suggests a multifactorial basis for obesity (Leibel, 2008; Day and Loos, 2011; Loos, 2012). Genes expressed in adipose tissue and with functional roles in adipogenesis, lipid turnover, the mitochondrion and energy expenditure and endocrine function might be of particular interest (Dahlman and Arner, 2007).

Linking energy restriction induced metabolic adaptations to genetic variants will give further insight in the heritability of inter-individual variation in susceptibility to weight regain and underlying mechanisms. The aim of this study was to examine the associations of the six selected single nucleotide polymorphisms (SNPs) with REE and physical activity before and after weight loss in overweight and obese subjects. In this study, we tested the individual effects of six genetic variants, which had shown associations with obesity-related traits from different angles before like physical activity (rs2076168 of peroxisome proliferator-activated receptorδ (*PPARD*) gene), fat distribution (rs1801282 of peroxisome proliferator-activated receptorγ2 (*PPARG2*) gene; rs1042713 of β2-adrenergic receptor (*ADRB2*) gene), regulation of energy expenditure (rs8192678 of peroxisome proliferator-activated receptorγ coactivator-1α (*PPARGC1A*) gene) and energy intake (rs9939609 of fat mass and obesity associated (*FTO*) gene; rs17782313 of melanocortin 4 receptor (*MC4R*) gene) (Gielen et al., 2014; Wheeler et al., 2013; Corella et al., 2012; Berglund et al., 2014; Beckers et al., 2011; Stefan et al., 2007; Petr et al., 2018; Canto and Auwerx, 2009; Grygiel-Gorniak, 2014; Loktionov, 2003; Tao, 2005). More specifically, *PPARD* and *PPARGC1A* have been associated with muscle morphology, oxygen uptake, power output and endurance performance (Petr et al., 2018) and in addition *PPARGC1A* is expressed in tissues with high energy oxidative capacity like the heart and brown adipose tissue (Canto and Auwerx, 2009); the *PPARG2* gene controls the expression of genes involved in adipocyte differentiation, fatty acid and glucose metabolism, and inflammatory processes (Grygiel-Gorniak, 2014); *ADRB2* is expressed in subcutaneous adipose tissue and it mobilizes lipids within human fat cells by stimulating lipolysis (Loktionov, 2003); *MC4R* is expressed in the brain and has been implicated in mediating the effects of melanocortin on food intake and energy expenditure (Tao, 2005) and *FTO* may be the best known genetic variant linked to obesity.

## 2 Materials and methods

### 2.1 Subjects

148 healthy subjects (109 women and 39 men), between 18 and 50 years old and with a body mass index (BMI) >28, were recruited by advertisements in local newspapers and on notice boards at the university. They underwent an initial screening that included measurement of body weight and height and the completion of a questionnaire on general health. All had to be in good health, not using medication (except for contraception), nonsmokers and at most moderate alcohol consumers. They were weight stable as defined by a weight change <5 kg for at least 3 months prior to the study. The study was conducted according to the guidelines laid down in the Declaration of Helsinki and procedures were approved by the Ethics Committee of the Maastricht University Medical Centre. Written informed consent was obtained from all participants. This trial was registered at clinicaltrials.gov as NCT01015508.

### 2.2 Study design

The study covered an 8-week period of a very low energy diet (VLED). Subjects had to come to the university on two occasions: the day before the start of the diet (baseline) and 8 weeks after the start of the diet (end of the diet). The protocol included measurement of REE and body composition from 08.00 in the morning onwards in the fasting state; only water was allowed in the twelve hours before the measurement. Two weeks prior to each measurement day, subjects received an accelerometer to measure physical activity over a two-week period. No physical activity instructions were given.

### 2.3 Diet

The weight loss diet (Modifast Intensive; Nutrition et Santé Benelux, Breda, The Netherlands) was followed for a period of 8 weeks. The diet was a protein-enriched formula that provided 2.1 MJ/day (51.9 g of protein, 50.2 g of carbohydrates and 6.9 g of lipids) and a micronutrient content, which meets the Dutch recommended daily allowance (Fernandes, 2002). The VLED was provided to the subjects as sachets with powder. Each sachet represented one meal and 3 sachets were consumed every day. Each subject received the same diet however in addition to the provided meal-replacements, they were allowed to eat vegetables when feeling hungry. Unlimited additional vegetables were allowed with the exception of starchy vegetables and legumes like potatoes or beans, which are relatively high in energy density. Subjects were instructed to mix the powder with the amount of water indicated on the packages and were advised to drink water sufficiently throughout the diet period. No dietary instructions were given in the two-week period before the baseline measurement.

### 2.4 Body composition

Height was measured at screening to the nearest 0.1 cm with the use of a wall-mounted stadiometer (model 220; Seca, Hamburg, Germany). Body composition was determined according to Siri's three-compartment model based on body weight, body volume and total body water. Body weight was measured using a calibrated scale (Life Measurement Corporation, Inc., Concord, CA, USA). Body volume was measured via air-displacement plethysmography with the BodPod System (Life Measurement Corporation, Inc., Concord, CA, USA) (Ginde et al., 2005; Dempster and Aitkens, 1995). Total body water was determined using deuterium dilution during the preceding night, according to the Maastricht protocol (Westerterp et al., 1995a). BMI was calculated by dividing body weight by height squared (kg/m^2^).

### 2.5 Resting energy expenditure

At 0800 h in the morning, subjects rested on a bed for 30 min, followed by 30 min of measuring their REE in the supine position using an open-circuit ventilated hood-system (Adriaens et al., 2003). Gas analyses were performed with a paramagnetic oxygen analyzer (Servomex, type 1158, Crowborough, East Sussex, UK) and an infrared carbon dioxide analyzer (Servomex, type 1520, Crowborough, East Sussex, UK) while flow was kept at a constant rate of 80 l/min and additionally measured as described by Schoffelen et al. (Schoffelen et al., 1997). The within individual coefficient of variation for this system is 3.3% ± 2.1 (Adriaens et al., 2003). Calculation of REE from measured oxygen consumption and carbon dioxide production was based on Brouwer's formula (Brouwer, 1957).

In addition to measuring REE with the ventilated hood system (REEm), REE was predicted (REEp) with the equation: REEp (MJ/d) = 0.024 x fat mass (kg) + 0.102 × fat free mass × (kg) + 0.85 (Westerterp et al., 1995b). Since fat mass (FM) and fat free mass (FFM) are used to calculate REEp, the equation can be used independently for gender. Adaptive thermogenesis was calculated as REEm divided by REEp. A value above 1 indicates that the change of the measured REE is higher than what is expected based on the body composition; a value lower than 1 indicates measured REE is lower than what is expected based on the body composition.

### 2.6 Physical activity monitoring

Physical activity was monitored in two-week intervals using the previously validated DirectLife triaxial accelerometer (Philips Research, Eindhoven, The Netherlands). The device is small and lightweight and was carried at an elastic belt around the waist. Subjects were instructed to wear the accelerometer during waking hours, except during showering and water activities. A diary was used to report periods in which the subject was not wearing the accelerometer during the day. The accelerometer output was processed to determine body movement by measuring activity counts. Total activity counts were calculated over the two-week monitoring period, and the sum of counts was divided by the number of monitoring days to determine the average activity counts per day (Bonomi et al., 2010; Bonomi et al., 2009).

### 2.7 DNA isolation and genotyping

Blood was collected in an EDTA tube during screening and the buffy coat was stored at −80 °C. Genomic DNA was isolated from the buffy coat using the QIAamp mini blood kit (Qiagen, Amsterdam, The Netherlands). Six SNPs that were associated earlier with relevant phenotypes, such as BMI or body composition, were selected and considered as indicators for genetic predisposition. These included rs9939609 of fat mass and obesity associated (*FTO*) gene; rs17782313 of melanocortin 4 receptor (*MC4R*) gene; rs1042713 of β2-adrenergic receptor (*ADRB2*) gene; rs1801282 of peroxisome proliferator-activated receptorγ2 (*PPARG2*) gene; rs8192678 of peroxisome proliferator-activated receptorγ coactivator-1α (*PPARGC1A*) gene; and rs2076168 of peroxisome proliferator-activated receptorδ (*PPARD*) gene.

Genotyping of five SNPs was performed using commercially available TaqMan SNP genotyping assays from Applied Biosystems (Foster City, California, USA). The procedure was performed according to the manufacturer's protocol and measured on an Applied Biosystems 7900 HT Fast Real-Time PCR system. Allelic calls were determined semi-automatically using the allelic discrimination software of Applied Biosystems. The C/G (Pro12Ala) polymorphism of the *PPARG2* gene was characterized using the polymerase chain reaction-restriction fragment length polymorphism (PCR-RFLP) assay. The primers used were 5′-GCCAATTCAAGCCCAGTC-3′ and 5′-GATATGTTTGCAGACAGTGTATCAGTGAAGGAATCGCTTTCCG-3′. The cycling conditions were 95 °C for 5 min, 30 cycles of 95 °C/30 s, 56 °C/45 s, 68 °C/45 s and followed by 68 °C for 7 min. The restriction enzym BstU-I was used, which generated the following fragments: 270 bp (CC); 270, 227, 43 bp (CG) and 227, 43 bp (GG).

### 2.8 Calculations and statistical analysis

First, a Chi-square test was used to check whether the allele frequencies were in Hardy Weinberg equilibrium. Second, repeated measures ANOVA was used to compare body composition, energy expenditure and physical activity parameters between 0 and 8 weeks with gender as covariate. Thirdly, mean baseline values of REEm, physical activity and REEm/REEp and short-term weight loss induced changes in REEm, physical activity and REEm/REEp were compared between the different genotypes within each of the 6 SNPs using general linear modelling. Bonferroni adjustment was used to correct for the multiple comparisons between the 3 genotypes within each SNP. Each SNP was tested individually, with age, gender, fat mass and fat free mass as covariates for the baseline parameters (unless otherwise indicated). Gender, age, weight loss (kg) and the baseline value for the tested parameter were used as covariates for the GLM when comparing the weight loss induced changes of the parameters between the genotypes. Spearman Rho's correlation coefficients were calculated for associations between parameters. Significance was defined as P < 0.05. Data were analyzed using SPSS 20.0 (SPSS, Inc., Chicago, IL, USA). Values are presented as mean and standard deviation (SD), unless otherwise indicated. The values resulting from the GLMs with Bonferroni correction are presented as adjusted means and their 95% confidence interval.

The power calculation was based on a weight loss study, in which a significant decrease in REE from 2068 to 1778 after 10% weight loss in obese subjects was found (Leibel et al., 1995b). With an α of 0.05 and β of 0.10 (power = 1-β = 0.90) the number of subjects needed is: N = [21 × (SD)^2^/(mean1-mean2)^2^] + 0.96 = 36. Taking into account a drop-out rate of 10% a total of 40 subjects is needed. Assuming a minor *FTO* allele frequency of 40% (den Hoed et al., 2009), at least 100 subjects were included.

## 3 Results

### 3.1 Body composition

After the 8 weeks of VLED, subjects (41 ± 9 years; BMI of 31.9 ± 3.0 kg/m^2^) lost on average 9.4 ± 4.1 kg (P < 0.001) consisting of 7.4 ± 3.3 kg of fat mass (FM) and 1.8 ± 2.2 kg of fat free mass (FFM) (Table 1). Subjects lost on average 10.5 ± 4.1% (P < 0.001) of the starting weight. FM decreased from 41.8 ± 6.3% to 37.4 ± 7.3% (P < 0.001). The data showed a large inter-individual variation in weight loss, indicating a difference in the success of weight loss. In addition, the variation in weight loss was not explained by different levels of physical activity at baseline or 8 weeks.

**Table 1:** Subject characteristics (mean ± standard deviation (standard error of the mean)) at baseline and after 8 weeks on a very low energy diet (n = 148).

	Pre-WL (0 weeks)	Post-WL (8 weeks)
Body weight (kg)	92.9 ± 12.2 (1.0)	83.5 ± 11.0^⁎⁎⁎^ (0.9)
BMI (kg/m^2^)	31.9 ± 3.0 (0.3)	28.9 ± 3.1^⁎⁎⁎^ (0.03)
Fat mass (kg)	38.7 ± 7.7 (0.7)	31.3 ± 7.9^⁎⁎⁎^ (0.07)
Fat free mass (kg)	54.0 ± 9.5 (0.8)	52.2 ± 9.0^⁎⁎⁎^ (0.08)
Percentage fat mass (%)	41.8 ± 6.3 (0.5)	37.4 ± 7.3^⁎⁎⁎^ (0.06)
REEm (MJ/d)	7.30 ± 1.02 (0.09)	6.65 ± 0.90^⁎⁎⁎^ (0.08)
REEp (MJ/d)	7.29 ± 0.98 (0.08)	6.92 ± 0.91^⁎⁎⁎^ (0.08)
REEp/REEm	1.00 ± 0.08 (0.01)	0.96 ± 0.08^⁎⁎^ (0.01)
Physical activity (Mcounts/d)	1.60 ± 0.38 (0.03)	1.56 ± 0.39^⁎^ (0.03)

BMI, body mass index; REEm, resting energy expenditure, measured; REEp, resting energy expenditure, predicted. ^⁎^ P < 0.05, ^⁎⁎^ P < 0.01, ^⁎⁎⁎^ P < 0.001.

### 3.2 Resting energy expenditure

REEm decreased significantly from 7.30 ± 1.02 MJ/d at baseline to 6.65 ± 0.90 MJ/d after the VLED (P < 0.001). The decrease of the REEm was expected, because body weight was reduced at all time points compared to baseline. Thus, the expected decrease in energy expenditure was observed in the predicted REE as well. REEp decreased significantly from baseline (7.29 ± 0.98 MJ/d) to after the VLED (6.92 ± 0.91 MJ/d, P < 0.001).

Comparing REEm with REEp at baseline, the ratio REEm/REEp was on average 1.00 ± 0.08. Values were highly correlated (R^2^ = 0.72; P < 0.001) and confirmed the validity of the prediction equation for the subject group under study. The ratio decreased significantly to 0.96 ± 0.08 after the VLED (P < 0.01).

### 3.3 Physical activity

Activity counts were on average 1.60 ± 0.38 Mcounts/d at baseline and decreased to 1.56 ± 0.39 Mcounts/d (P < 0.05) after 8 weeks of energy restriction.

### 3.4 Genetic predisposition

Genotypic and allelic distributions per single nucleotide polymorphism are shown in Table 2.

**Table 2:** Genotypic and allelic distributions per single nucleotide polymorphism.

Gene	SNP	G	F(N)	F(%)	Allele⁎	F(%)	HWE
*FTO*	rs9939609	AA	25	16.9	**A**	38.9	0.65
		AT	65	43.9	T	61.1	
		TT	58	39.2			
*MC4R*	rs17782313	CC	15	10.1	**C**	24.1	0.19
		CT	46	31.1	T	75.9	
		TT	87	58.8			
*ADRB2*	rs1042713	GG	56	37.8	G	62.3	0.92
		GA	71	48.0	**A**	37.7	
		AA	21	14.2			
*PPARD*	rs2076168	GG	16	10.9	**G**	28.1	0.33
		GT	52	35.4	T	71.9	
		TT	79	53.7			
*PPARGC1A*	rs8192678	AA	20	13.6	**A**	37.2	0.95
		AG	70	47.6	G	62.8	
		GG	57	38.7			
*PPARG2*	rs1801282	GG	1	0.7	**G**	10.7	0.83
		CG	30	20.1	C	89.3	
		CC	117	79.2			

G, genotype; F, frequency, both absolute (N) and relative (%).

P-values obtained from the χ^2^-test of Hardy Weinberg equilibrium (HWE).

⁎ Risk/minor allele in bold.

**Fig. 1:**
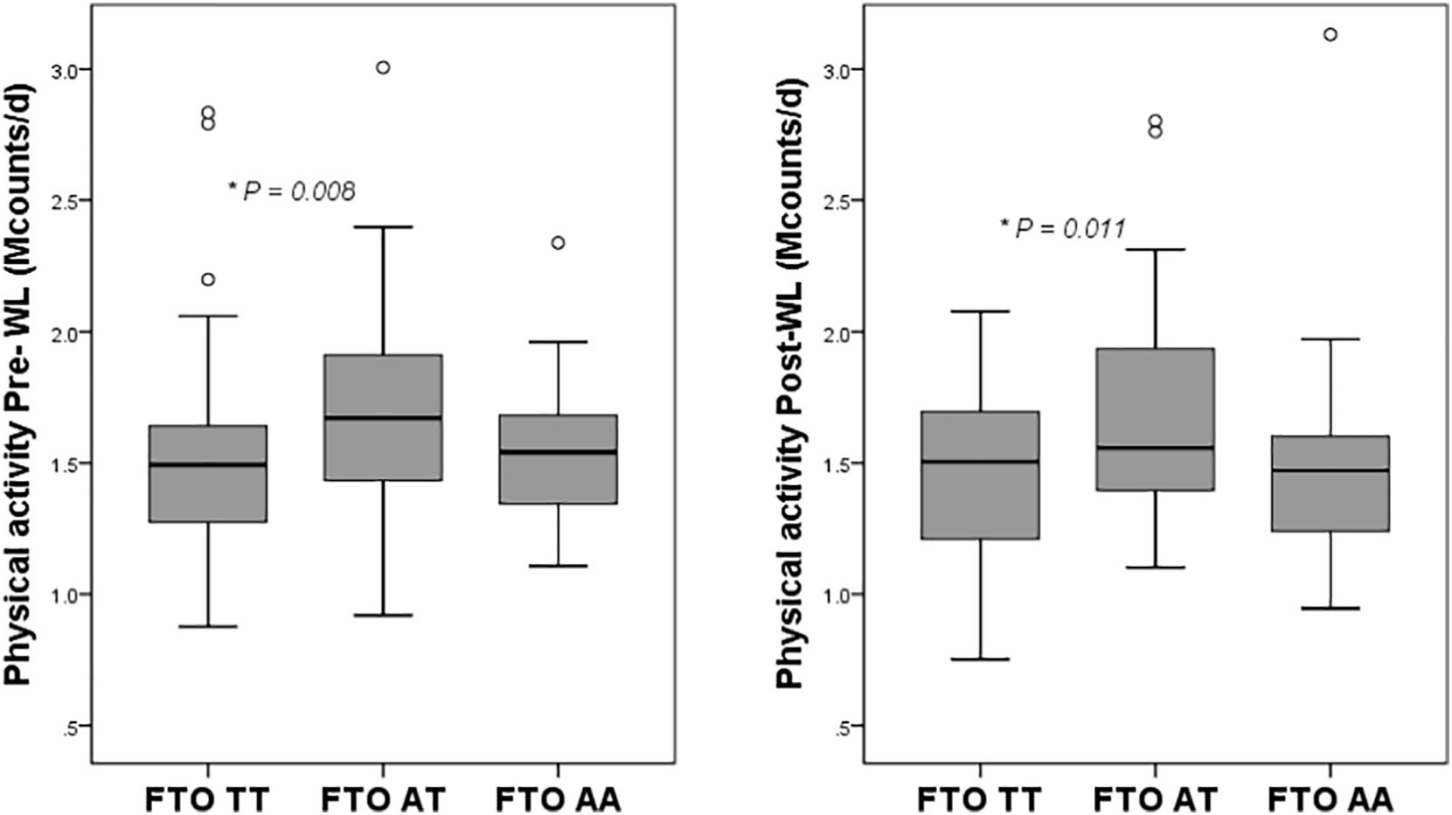
Boxplots of pre-WL physical activity on the left panel and post-WL physical activity on the right panel (Mcounts/d) according to *FTO* rs9939609 polymorphism genotypes groups, adjusted for age, gender, fat mass and fat free mass. Values are unadjusted mean values. * P < 0.01.

At baseline, the heterozygotic genotype (AT) of *FTO* was associated with a higher amount of physical activity (1.71 Mcounts/d; CI 1.62–1.81) compared to the homozygotic major genotype (TT) (1.50 Mcounts/d; CI 1.40–1.59) (P < 0.001) while the homozygotic minor genotype (AA) was not significantly different (1.56 Mcounts/d; CI 1.39–1.74) (Fig. 1). After the 8 weeks of VLED, a similar pattern was observed: subjects with the AT genotype (1.68 Mcounts/d; CI 1.58–1.78) showed a higher amount of physical activity compared to the TT genotype (1.49 Mcounts/d; CI 1.38–1.58) (P < 0.05) while subjects with the AA genotype showed no significant difference (1.52 Mcounts/d; CI 1.34–1.70) (Fig. 1). No differences were observed between the *FTO* genotypes for the change in physical activity over 8 weeks.

**Fig. 2:**
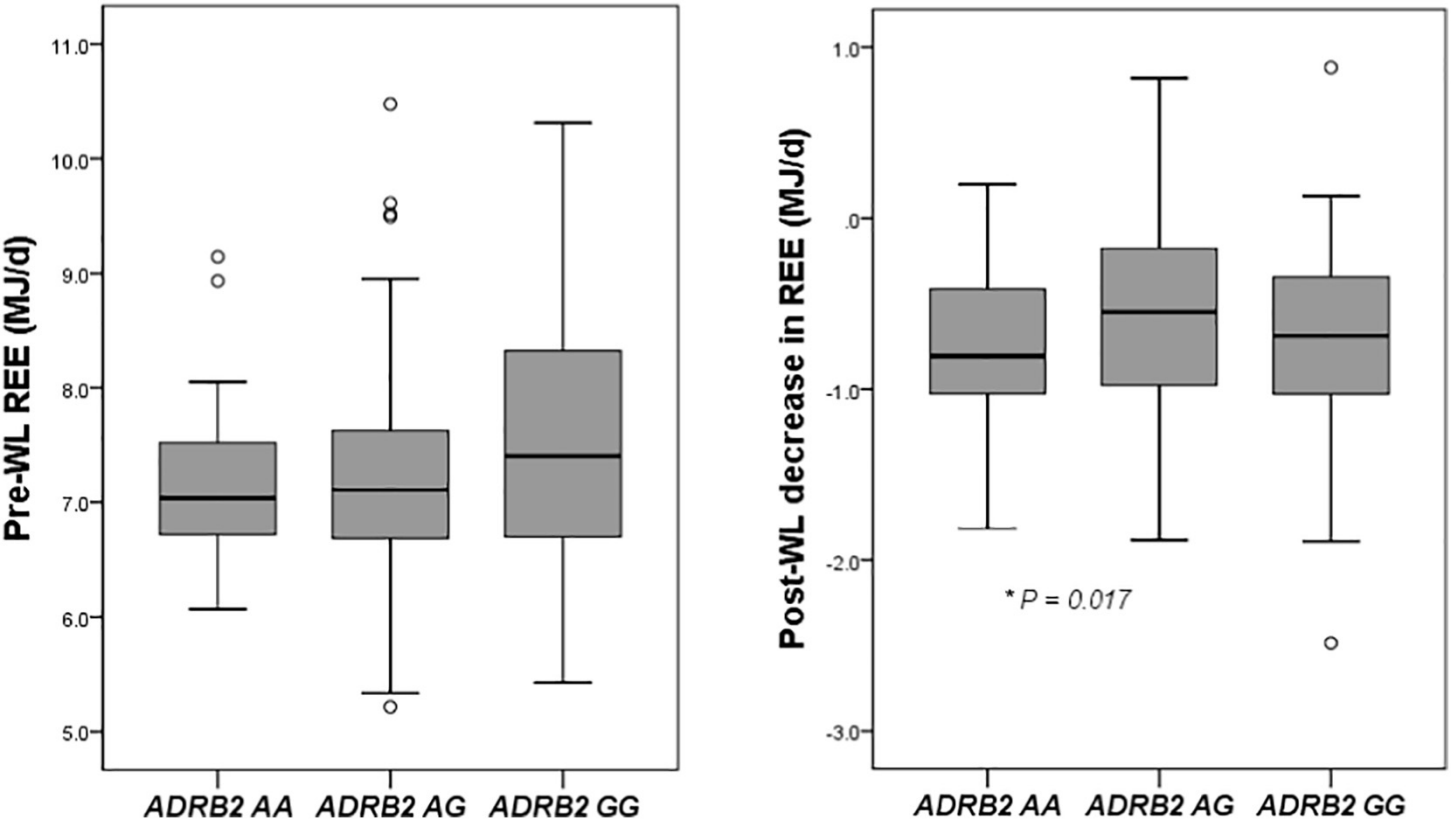
Boxplots of baseline resting energy expenditure on the left panel and change in REE after 8 wks on the right panel (MJ/d) according to *ADRB2* rs1042713 polymorphism genotypes. Adjusted for age, gender, fat mass and fat free mass (left), for age, gender, weight loss (kg) and baseline REE (right). Values are unadjusted mean values * P < 0.05.

Subjects homozygous for the minor *ADRB2* allele (AA) were linked to a lower REEm (7.11 MJ/d; CI 6.86–7.35) compared to subjects with the heterozygotic genotype (AG) (7.42 MJ/d; CI 7.27–7.57) (P = 0.1), while no difference was observed with subjects homozygous for the common allele (GG) (7.29 MJ/d; CI 7.11–7.48) (Fig. 2). After weight loss, a larger decrease in REE was associated with carrying the homozygotic minor genotype of *ADRB2* (AA) (−0.90 MJ/d; CI −1.10- −0.71) compared to subjects with the AG genotype (−0.57 MJ/d; CI −0.69- −0.45) (P < 0.05), carriers of the GG phenotype were not significantly different (−0.67 MJ/d; CI −0.82- −0.53) (Fig. 2).

**Fig. 3:**
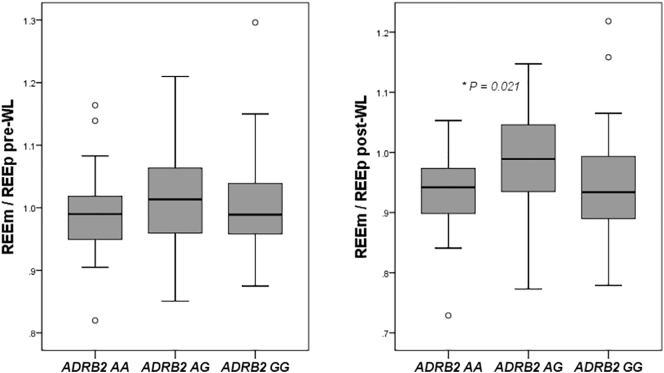
Boxplots of measured resting energy expenditure (REEm) divided by the predicted resting energy expenditure (REEp) before WL on the left panel and after weight loss on the right panel according to *ADRB2* rs1042713 polymorphism genotypes. Adjusted for age, gender, fat mass and fat free mass (left), for age, gender, weight loss (kg) and baseline REEm/REEp (right). Values are unadjusted mean values *P < 0.05.

At baseline no differences for REEm/REEp were observed between the three genotypes of *ADRB2*. However after weight loss, subjects homozygous for the minor *ADRB2* allele (AA) were associated with a lower REEm/REEp after weight loss (0.936; CI 0.909–0.963) compared to carriers of the heterozygotic genotype (AG) (0.981; CI 0.964–0.998) (P < 0.05) while carriers of the GG phenotype were not significantly different (0.959; CI 0.938–0.980) (Fig. 3). The change in REEm/REEp during weight loss was greater in subjects that were homozygous for the AA allele (−0.077; CI −0.108- −0.46) (P < 0.005) and in subjects that were homozygous for the GG genotype (−0.056; CI −0.078- −0.034) (P < 0.05) compared to carriers of the AG genotype (−0.17; CI -0.036-0.003) (Fig. 3).

No differences were found between genotype variations in *MC4R*, *PPARG2*, *PPARGC1A*, or *PPARD* and REE, physical activity as well as energy restriction induced adaptations in REE and physical activity.

## 4 Discussion

The present study shows that obese men and women homozygous for the minor *ADRB2* rs1042713 allele (AA) show a larger energy restriction induced adaptation in REE or adaptive thermogenesis. Furthermore, subjects carrying the heterozygotic genotype of *FTO* rs9939609 had higher physical activity at baseline and this was carried over after weight loss.

Results from the current study showed an association between the minor *ADRB2* allele and lower REE but showed no association between REE and *FTO* genotype variation. This is in line with previous results in adult humans (Berentzen et al., 2008; Speakman et al., 2008; Do et al., 2008), except for one study that did find lower REE in women carrying the minor allele of *FTO* (Arrizabalaga et al., 2013). Moreover, obese men and women homozygous for the minor and common *ADRB2* allele showed a greater adaptation in the REE induced by energy restriction, often referred to as adaptive thermogenesis (Camps et al., 2013). The disproportionate reduction in energy expenditure favors a positive energy balance following weight loss and may predispose to weight regain (Camps et al., 2013; Tremblay et al., 2013). Previously, several studies have suggested a link between the *ADRB2* risk allele and the metabolic syndrome (Dallongeville et al., 2003; Park et al., 2008), whereas Ruiz et al. showed higher weight loss in women carrying the common *ADRB2* allele (Ruiz et al., 2011). The *ADRB2* receptor plays a role in the regulation of lipolysis (Rosado et al., 2015). *ADRB2* is a member of the G-protein-coupled adrenergic receptor superfamily and participates in the peripheral regulation of energy expenditure (Loktionov, 2003). *ADRB2* is expressed in subcutaneous adipose tissue and it mobilizes lipids within human fat cells by stimulating lipolysis. Experimental studies have shown that the minor *ADRB2* allele reduces the function of the β2-receptor. The association between REE, adaptive thermogenesis, and the altered function of the β2-adrenergic receptor, suggest that a reduced ability to mobilize lipids within adipose tissue through lipolysis to compensate for reduced energy intake has a central role in the metabolic adaptations. This seems to be in line with results from a previous study, which showed that people with a reduced ability to increase β-oxidation of free fatty acids had greater energy restriction induced thermogenesis (Camps et al., 2015). Furthermore, Bouwman et al. showed a positive correlation between three enzymes of the beta-oxidation (HADHsc, Acetyl-CoA Acetyltransferase and Acyl-CoA dehydrogenase) and plasma free fatty acids (Bouwman et al., 2009). Together these results indicate that a better ability to up-regulate beta-oxidation during energy restriction, and an increased ability to mobilize free fatty acids into the circulation may lead to less adaptive thermogenesis. Recently, Ramos-Lopez et al. showed that *ADRB2* polymorphisms influenced blood lipid outcome depending on the type of dietary intervention, further indicating the importance of nutrigenetic understanding in weight management (Ramos-Lopez et al., 2018).

Higher physical activity was found in subjects carrying the heterozygotic genotype, one risk allele of the *FTO* polymorphism and one common allele (AT), compared to subjects that were homozyguous for the common allele (TT). Subjects carrying the homozygotic risk allele (AA) showed similar levels of physical activity as the TT genotype, however their number was too low to show a significant difference with the more active heterozygotic genotype. Previously, studies have shown an association between the *FTO* risk allele and a higher body weight and BMI (Scuteri et al., 2007; Verhoef et al., 2014), and people with a higher BMI tend to be less physical active (Ekelund et al., 2002). The current results are in line with previous studies and suggest that the increased risk for obesity associated with the *FTO* risk allele are not associated with reduced physical activity (Loos and Yeo, 2014; Kilpelainen et al., 2011). Our results suggests that homozygotic carriers of the *FTO* risk allele have similar levels of physical activity compared to homozygotic carriers of the common allele. This is in line with a 2011 meta-analysis on data of 218,166 adults and 19,268 children that showed that the increased risk of obesity due to genetic variation in *FTO* can be attenuated by physical activity, but did not find an association between *FTO* variation and a decreased level of physical activity (Kilpelainen et al., 2011).

Besides the known relationship between *FTO* and BMI, weight, and energy expenditure (Scuteri et al., 2007; Verhoef et al., 2014; Ekelund et al., 2002), it is known that carriers of the *FTO* risk allele have an increased food intake between 0.50 and 1.23 MJ/d compared to common *FTO* allele carriers. Furthermore, the BMI-increasing allele of *FTO* has been associated with a higher intake of fat and protein, reduced satiety, worse food choices and bad eating habits and lower control over eating (Loos and Yeo, 2014). Possible mechanisms have mainly pointed towards increased expression of *FTO* in the hypothalamic site of brain tissue which controls food intake (Zhao et al., 2014). The current results showed an increased level of physical activity in subjects carrying one *FTO* risk allele, which might partly explain the lower weight compared to homozygotic carriers of the risk allele (Verhoef et al., 2014). Although the mechanism is not clear, these and previous results show clearly that the increased risk of carrying the *FTO* risk allele can be in part mitigated by increased physical activity.

In the current study we did not observe associations between genotype variations in *MC4R*, *PPARG2*, *PPARGC1A*, or *PPARD* and REE and physical activity before and after weight loss. For *MC4R*, the lack of an association is in accordance with previous results that showed no influence of the *MC4R* rs17782313 polymorphism on energy metabolism in obese women (Arrizabalaga et al., 2013). Arrizabalaga et al. suggested that the genetic effects of the MC4R gene are possibly more pronounced during childhood (Arrizabalaga et al., 2013). As far as we are aware, studies determining the relation between *PPARG2*, *PPARGC1A*, or *PPARD* genotypes and REE, physical activity as well as energy restriction induced adaptations are very limited. The absence of any observed associations between variations in the PPAR genes with adaptive thermogenesis and decreased total physical activity suggests that the *PPAR* genes may not play a major role in the regulation of REE and total physical activity during energy restriction. It is known that *PPARD* and *PPARGC1A* expression are associated with muscle tissue, oxygen uptake, power output and endurance performance (Petr et al., 2018), though this does not necessarily equate to the amount of total physical activity during energy restriction. It should be noted that the absence of any significant associations in the current study does not exclude that the differences might have been too small to detect or a greater number of subjects would have been required to detect it.

From the current results we cannot determine whether there were gene-environment interactions masking effects of a genetic variant. In addition, six SNPs as measured in 148 subjects are low compared to other genetic studies. However, this is a consequence of our study design since accurate assessment of REE, physical activity and adaptations during short-term weight loss was a limiting factor. A recent study suggests that *IRX3* is a functional long-range target of obesity-associated variants within *FTO*, and represents a novel determinant of body mass and composition; however, they do not dispute the idea emerging from animal studies that *FTO* is also a regulator of body mass and composition (Smemo et al., 2014). As a result of the current study design, the energy deficit is not equal for all subjects. However, in this study, weight loss was not a primary objective but a mediator to induce adaptations in energy expenditure and physical activity which were the primary outcome parameters. Furthermore, a larger range in weight loss resulted in a larger range of metabolic adaptations to examine in relationship to variants in the selected genes.

In conclusion, carrying the minor *ADRB2* allele homozygous was associated with a larger diet induced metabolic adaptation in energy expenditure. The association between REE, adaptive thermogenesis and the altered function of the β2-adrenergic receptor, suggests that a reduced ability to mobilize lipids from adipose tissue through lipolysis as an energy source to compensate for reduced energy intake, might play a central role in the metabolic adaptations in energy expenditure during weight loss. In line with literature, carrying the risk allele of *FTO* homozygous was not associated with lower physical activity at baseline or after weight loss. However, heterozygous carriers of one risk allele showed higher physical activity before and after weight loss which might protect them in part from the higher risk associated with *FTO*.

## Disclosure statement

The authors have nothing to disclose.

## CRediT authorship contribution statement

**Stefan G.J.A. Camps:** Formal analysis, Investigation, Methodology, Project administration, Resources, Software, Validation, Visualization, Writing - original draft, Writing - review & editing. **Sanne P.M. Verhoef:** Formal analysis, Investigation, Methodology, Project administration, Resources, Software, Validation, Writing - review & editing. **Freek G. Bouwman:** Methodology, Software, Validation, Writing - review & editing. **Edwin C.M. Mariman:** Supervision, Validation, Writing - review & editing. **Klaas R. Westerterp:** Conceptualization, Funding acquisition, Supervision, Validation, Writing - review & editing.
